# Effect of a short-term vitamin E supplementation on oxidative stress in infertile PCOS women under ovulation induction: a retrospective cohort study

**DOI:** 10.1186/s12905-020-00930-w

**Published:** 2020-04-06

**Authors:** Jie Chen, Qian Guo, Ying-hao Pei, Qing-ling Ren, Lei Chi, Rong-kui Hu, Yong Tan

**Affiliations:** 1 Departments of Gynaecology, Jiangsu Province Hospital of Chinese Medicine, Affiliated Hospital of Nanjing University of Chinese Medicine, Nanjing, China; 2 Departments of Intensive Care Unit, Jiangsu Province Hospital of Chinese Medicine, Affiliated Hospital of Nanjing University of Chinese Medicine, Nanjing, China; 3 Departments of Reproduction, Jiangsu Province Hospital of Chinese Medicine, Affiliated Hospital of Nanjing University of Chinese Medicine, Nanjing, 210046 China

**Keywords:** Vitamin E, Polycystic ovary syndrome, Ovulation induction, Infertility

## Abstract

**Background:**

Vitamin E, which is critically important in the whole process of reproduction, can antagonize the oxidative stress caused by the oxygen free radicals and antioxidant imbalance and regulate normal physiological function of the reproductive system. The effect of short-term supplementation of vitamin E on outcomes of infertile women with polycystic ovary syndrome (PCOS) when they underwent ovulation induction with clomiphene citrate (CC) and human menopausal gonadotropin (HMG) remains unknown.

**Methods:**

This was a retrospective cohort clinical trial from October 2015 to April 2017. A total of 321 PCOS cases underwent ovulation induction with CC and HMG. Patients in group A (*n* = 110) did not receive vitamin E while patients in group B (*n* = 105) and group C (*n* = 106) received oral treatment of vitamin E at 100 mg/day during follicular phase and luteal phase, respectively.

**Results:**

It was observed no significant differences of ovulation rate, clinical pregnancy rate, and ongoing pregnancy rate among the three groups. It was interesting that dosage of HMG were significant lower in group B compared with those in group A and group C (P<0.05).

**Conclusions:**

A short-term supplementation of vitamin E can improve oxidative stress, and reduce exogenous HMG dosage to lower the economic cost with a similar pregnancy rate in the ovulation induction cycle. However, the supplementation does not alter the pregnancy rate in the ovulation induction cycle.

**Trial registration:**

ChiCTR-OOC-14005389, 2014.

## Background

Polycystic ovary syndrome (PCOS) was initially described by Stein and Leventhal in 1935, with a 10%-prevalence in women of reproductive age [1, 2]. We generally recognize four key features of PCOS: (1) ovulatory and menstrual dysfunction, (2) hyperandrogenemia, (3) clinical features of hyperandrogenism, and (4) polycystic ovaries [3]. PCOS is a complex process and the underlying mechanism remains poorly understood. Oxidative stress, defined as an imbalance between pro- and anti-oxidants [4], has been recognized to be involved in the development of PCOS [5, 6]. PCOS is demonstrated to be accompanied with a state of oxidative stress and inflammation, strongly associated with insulin resistance irrespective of obesity [7] and defective follicle maturation [8].

Vitamin E as a lipid-soluble substance with non-enzymatic antioxidant properties, also known as tocopherol, was found for the first time by Evans and Bishop in 1922 [9]. Vitamin E can effectively reverse the adverse influence by oxidative stress brought to the reproductive system and endocrine system, and is widely used in the field of reproductive medicine. Vitamin E, which is critically important in the whole process of reproduction, can antagonize the oxidative stress caused by the oxygen free radicals and antioxidant imbalance through inhibiting the activity of phospholipase A and lipoxygenase to stabilize cell membrane, and regulate normal physiological function of the reproductive system. With the anti-oxidant properties, vitamin E can reduce the senile oxidative stress reaction that may have a detrimental effect on the number and quality of oocytes [10]. A lack of vitamin E can lead to female infertility, miscarriage, premature delivery, eclampsia, fetal intrauterine growth restriction and other diseases associated with pregnancy [11–13] and abnormal semen quality [14].

Recent evidence has clearly confirmed the benefit of ovulation induction with clomiphene citrate (CC) and human menopausal gonadotropin (HMG) in PCOS women [15, 16]. The aim of this study was to determine if short-term supplementation of vitamin E would lead to improved reproductive performance in ovulation induction for PCOS and to explore the associations between vitamin E and pregnancy rates.

## Methods

### Study design

The present study is a retrospective cohort study (Trial registration: ChiCTR-OOC-14005389, 2014). In this study, 321 PCOS cases was conducted from October 2015 to April 2017 to assess the effect of short-term vitamin E administration on infertile PCOS women undergoing ovulation induction with CC and HMG in the Reproductive Medicine Center, Jiangsu Province Hospital of Chinese Medicine, Nanjing, China. This retrospective cohort study was approved by the Institutional Review Board of the Department of Chinese Medicine Hospital of Jiangsu Province.

### Study population

The inclusion criteria of this study were as follows: (i) Undergoing ovulation induction with CC and HMG; (ii) no previous infertile treatment; (iii) age less than 40 years; (iv) normal in hysterosalpingography; and (v) normal in semen analysis. The diagnostic criteria of PCOS was according to the 2006 Rotterdam criteria [17]: (1) Anovulation or olig-ovulation, (2) Clinical evidence of hyperandrogenism (on the basis of hirsutism or an elevated testosterone level), (3) Polycystic ovaries (a more than 10 ml ovarian volume or at least 12 antral follicles with 2–9 mm in diameter). PCOS could be confirmed if any 2 out of the following 3 criteria were met and if any other diseases that caused hyperandrogenism or anovulation could be excluded.

Other disorders that mimic the PCOS, including hyperprolactinemia, thyroid disease, late-onset congenital adrenal hyperplasia, androgen-secreting tumors and Cushing’s syndrome were ruled out. The PCOS patients with the major myocardial, liver and renal disorders, and taking confounding medications (primarily sex steroids, other infertility drugs, and insulin sensitizers) were excluded. Patients were divided into 3 groups according to the vitamin E used.

### Vitamin E administration

**Fig. 1:**
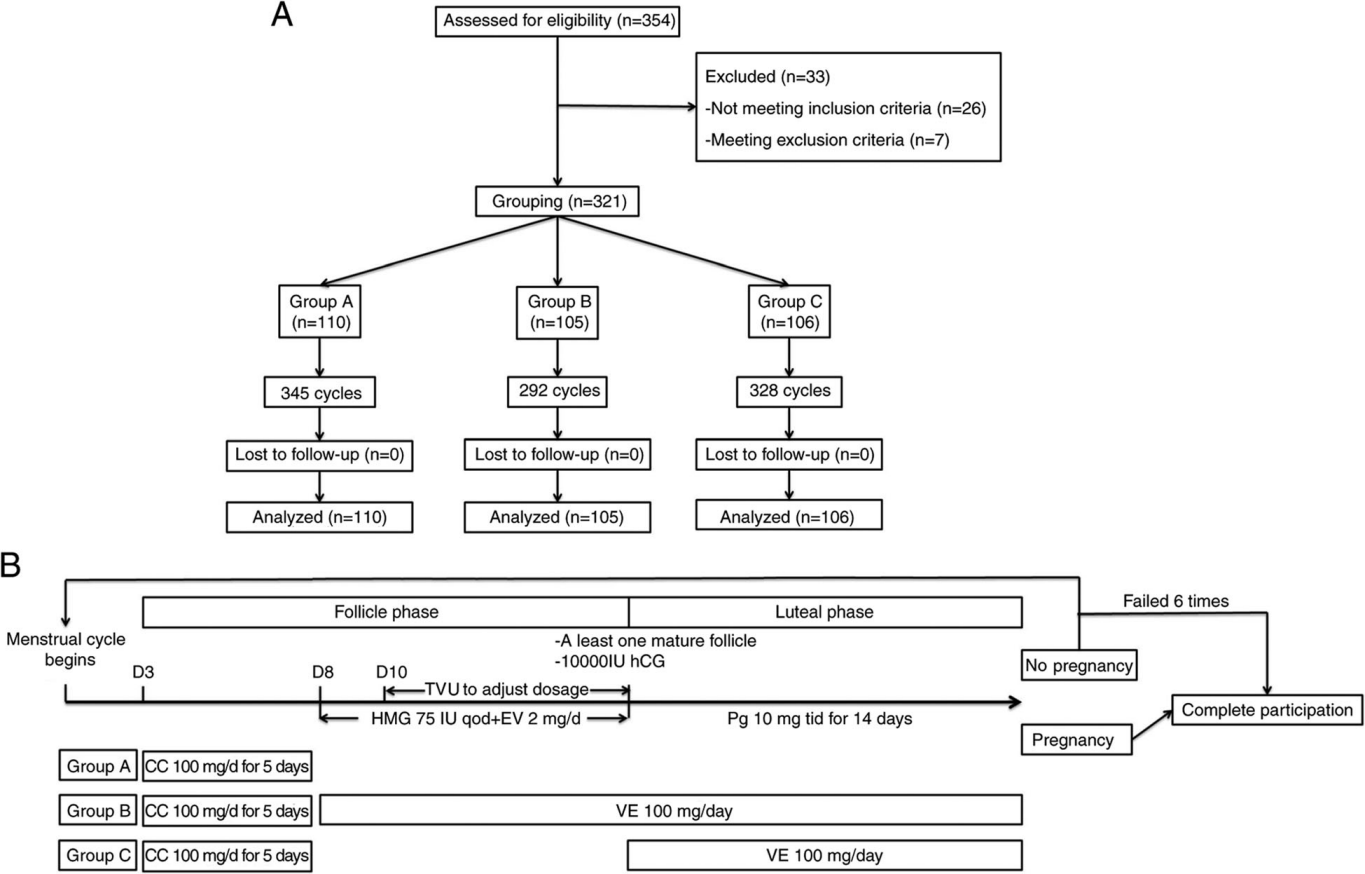
Summary of patient flow diagram **a** and Vitamin E administration and stimulation protocol **b**. EV = estradiol valerate; HCG = urinary human chorionic gonadotropin; Pg = progesterone;CC = clomiphene citrate;VE = vitamin E;TVU = Transvaginal ultrasonography; HMG = human menopausal gonadotropin

In this study, as shown in Fig. 1a, 110 of 321 PCOS cases underwent controlled ovarian stimulation but without vitamin E administration (Group A, *n* = 110). Based on previous clinical medication experience, a dosage of 100 mg/day vitamin was selected. Two-hundred eleven of or 321 PCOS cases underwent controlled ovarian stimulation combined with vitamin E administration (100 mg/day, p.o.) started from follicular phase (Group B, *n* = 105) and luteal phase (Group C, *n* = 106), respectively. Administration of vitamin E in follicular phase(Group B) began from the 3rd day of the menstrual cycle to 14th day of luteal phase. Administration of vitamin E in luteal phase (Group C) started when ovulation was confirmed, and lasted for 14 consecutive days. After 14 days of the HCG administration, serum β-HCG was measured. The presence of a gestational sac on ultrasound was performed at 6 and 12 weeks of gestational age to determine clinical pregnancy rate and ongoing pregnancy. The women enrolled in this study were followed up until miscarriage or delivery.

### Stimulation protocol

As shown in Fig. 1b, the ovulation was stimulated with CC (Merck Serano, China) at 100 mg/day for 5 days starting on day 3 of a spontaneous menstrual cycle or withdrawal bleeding. Starting from day 8, HMG (Livzon, China) was injected at 75 IU every second day and estradiol valerate (Progynova, Bayer, China) was administered at 2 mg/day. Transvaginal ultrasonography was performed from day 10 to adjust the HMG dosage. When at least one follicle had reached a diameter of 18 mm, 10,000 IU urinary human chorionic gonadotropin (hCG) (Livzon, China) was administered. All patients received luteal phase support by oral administration of progesterone (Dydrogesterone, Abbott Biologicals B.V, China) at 10 mg three time a day for 14 days starting on the day of ovulation. In each cycle, medroxyprogesterone acetate was used to induce withdrawal bleeding in cases in which there was no response. The complete participation considered as pregnancy or anovulation within a total of 6 cycles.

Body mass index (BMI) was used to evaluate the body weight. According to World Health Organization(WHO) criteria [18], women with a BMI < 18.5, 18.5–25, 25–29 and ≥ 30 kg/m^2^ were defined as underweight, normal weight, overweight and obese, respectively. Scores on the modified Ferriman–Gallwey scale [19], range from 0 to 36, were used for hirsutism evaluation. Higher scores indicated a greater degree of hirsutism.

### Data collection

#### Baseline data

Age, height, weight, waist, Ferriman–Gallwey hirsutism score, age of menarche, incidence of oligomenorrhea and amenorrhea, numbers of previous pregnancies and previous ovarian were obtained from patient medical records.

#### Hormonal analyses

Levels of estradiol (E_2_), androstenedione (T), luteinizing hormone (LH), prolactine (PRL) and follicle-stimulating hormone (FSH) were tested by RIA (Beijing North Institute of Biological Technology of China and the CIS Company of France). Peripheral blood samples were taken on the 3th day of menstrual cycle after overnight fasting.

### Oxidative stress evaluation

In this study, we measured four oxidative stress serum markers (malondialdehyde (MDA), ischemia modified albumin (IMA), total antioxidant capacity measurements (TAC), and vitamin E) in 3 time points (T0:before stimulation, T1: the day of HCG treatment, and T2: the day of complete participation) to evaluate the levels of oxidative stress. Serum levels of MAD, an end-product formed during lipid peroxidation that is released into the extracellular space and finally appears in the blood [20], were measured using a thiobarbituric acid-reactive commercial kit (Jiancheng Bioengineering Institute, China). Serum TAC, provided better information on antioxidant status than individual antioxidant compounds [21], were tested using an antioxidant assay kit (Jiancheng Bioengineering Institute, China). Serum levels of IMA, a novel marker of oxidative stress, were evaluated by cobalt to albumin binding capacity kit (CUSABIO, China). Serum contents of vitamin E were evaluated by colorimetric method using assay kit (Jiancheng Bioengineering Institute, China).

### Statistical analysis

Statistical analysis was carried out by SPSS (version 23, USA). Data were presented as either median (Min-Max) or mean ± SD as appropriate. Quantitative data analyses were carried out by independent samples *t*-test or Mann-Whitney U-test depending on the normality of data. Categorical variables were compared with Chi-Square test. A *P* value < 0.05 was considered as statistically significant.

## Results

### Patient baseline data

The characteristics of enrolled PCOS women are summarized in Table 1. A significant statistical difference was not observed among the three groups with respect to age, age of menarche, BMI, obesity parameters, Ferriman-Gallwey hirsutism score, prevalence of menstrual patterns, the duration of infertility, number of previous pregnancies, number of previous ovarian stimulation and levels of base sex hormones. Most of the women in this study were normal weight or overweight, as also shown in Table 1, 70(63.6%), 68(64.7%) and 65(61.4%) in group A, B and C, respectively.

**Table 1:** The characteristics and basal serum sex hormones of PCOS women

	Group A *n* = 110	Group B *n* = 105	Group C *n* = 106
Age (years)	25.87 ± 2.63	26.88 ± 2.84	26.81 ± 2.64
Age of menarche (years)	14.09 ± 1.43	14.62 ± 1.48	14.40 ± 1.71
BMI (kg/m^2^)	22.91 ± 4.05	23.80 ± 3.69	23.57 ± 3.89
< 18.5	8(7.3)	7(6.7)	5(4.7)
18.5- < 25	55(50)	52(49.5)	52(49.1)
23- < 29	15(13.6)	16(15.2)	13(12.3)
≥ 30	32(29.1)	30(28.6)	36(33.9)
Waist(cm)	76.3 ± 14.3	74.1 ± 11.9	74.9 ± 12.2
WHR	0.89 ± 0.08	0.87 ± 0.07	0.89 ± 0.06
Ferriman–Gallwey hirsutism score	7.54 ± 3.36	7.15 ± 2.99	7.09 ± 3.10
Menarche(year)	12.9 ± 2.8	13.2 ± 2.0	13.1 ± 2.9
Oligomenorrhea(*n*, %)	56(50.9)	53(50.5)	55(51.9)
Amenorrhea (*n*, %)	54(49.1)	52(49.5)	51(48.1)
Infertility duration (years)	2.16 ± 0.56	2.02 ± 0.51	2.21 ± 0.67
Previous live birth (*n*, %)	21(19.1)	19(18.1)	22(20.8)
No. of previous pregnancies	0.60 ± 0.75	0.47 ± 0.80	0.42 ± 0.54
No. of previous ovarian stimulation	0.89 ± 0.78	0.91 ± 1.17	1.07 ± 1.06
E_2_ (pg/mL)	44.47 ± 28.87	44.87 ± 30.52	45.61 ± 37.42
T (nmol/L)	1.33 ± 0.59	1.49 ± 0.52	1.51 ± 0.58
LH (mIU/mL)	6.94 ± 3.21	7.44 ± 3.45	6.85 ± 2.82
FSH (mIU/mL)	5.30 ± 1.67	5.29 ± 2.35	5.43 ± 2.44
PRL (ug/L)	14.24 ± 7.92	14.97 ± 9.97	14.76 ± 8.01

Data is shown as means ± SD. *Group A* treated without vitamin E, *Group B* treat with vitamin E in follicular phase, *Group C* treat with vitamin E in luteal phase, *WHR* waist to hip ratio, *E*_*2*_ estradiol, *T* testosterone, *LH* luteinizing hormone, *FSH* follicle-stimulating hormone, *PRL* prolactin

### The outcomes of PCOS women in ovulation induction with or without vitamin E

On the whole, 965 cycles were studied in 321 patients:345 cycles in group A (110 patients), 292 cycles in group B (105 patients) and 328 cycles in group C (106 patients). Dosage of HMG in group B were significanlty lower than those in group A and C(*P*<0.05). On day of HCG administration, endometrium thickness, mean total E2 level and mean E2 level per mature follicle in group B were higher than those in group A and C(*P* < 0.05). No statistical significance existed in the duration of stimulation and number of dominant follicles (≥18 mm) in each groups (*P* > 0.05). The ovulation rate, clinical pregnancy rate, and ongoing pregnancy rate in each group did not show significant difference (*P* > 0.05) (Table 2). The ovulation rate showed no significant difference between obese and non-obese patients in each group (*P* > 0.05) (Table 3).

**Table 2:** The outcomes of PCOS women in ovulation induction with or without vitamin E

	Group A (*n* = 110)	Group B (*n* = 105)	Group C (*n* = 106)
Total cyclc	345	292	328
HMG dosage (75 IU/bottle)	6.62 ± 2.37	5.29 ± 2.35^*△^	6.43 ± 2.44
No. of dominant follicles (≥18 mm)	1.44 ± 0.89	1.30 ± 0.74	1.50 ± 0.88
Data on day of HCG administration			
Endometrium thickness (mm)	7.38 ± 1.43	8.33 ± 1.19^*△^	7.76 ± 2.03
Mean total E2 (pg/ml)	245.23 ± 126.74	336.51 ± 155.62^*△^	214.92 ± 114.11
Mean E2 per mature follicle(pg/ml)	162.55 ± 60.40	261.81 ± 92.88^*△^	146.61 ± 88.51
Ovulation rate	85 (77.3%)	75 (71.43%)	78 (73.6%)
No. of ovulations/total cycles	85/345(24.6%)	75/292(25.7%)	78/328(23.8%)
Clinical pregnancy rate	31 (28.2%)	29 (27.6%)	25 (23.6%)
Ongoing pregnancy rate	27 (24.5%)	29 (27.6%)	24 (22.6%)

Data is shown as means ± SD. ^*^ Compared with group A, P<0.05. ^△^Compared with Group C, P<0.05. Group A: treated without vitamin E, Group B: treat with vitamin E from follicular phase, Group C: treat with vitamin E from luteal phase

**Table 3:** Comparisions of ovulation rates between obese(BMI ≥ 30 kg/m^2^) versus non-obese(BMI < 30 kg/m^2^) women

	Obese	Non-obese
CC alone	21/32(65.6)	64/78(82.1)
CC + Vit E	45/66(68.2)	108/145(74.5)
Total	66/98(67.3)	172/223(77.1)

*CC* clomiphene citrate

### Serum oxidative stress of PCOS women in ovulation induction with or without vitamin E

**Fig. 2:**
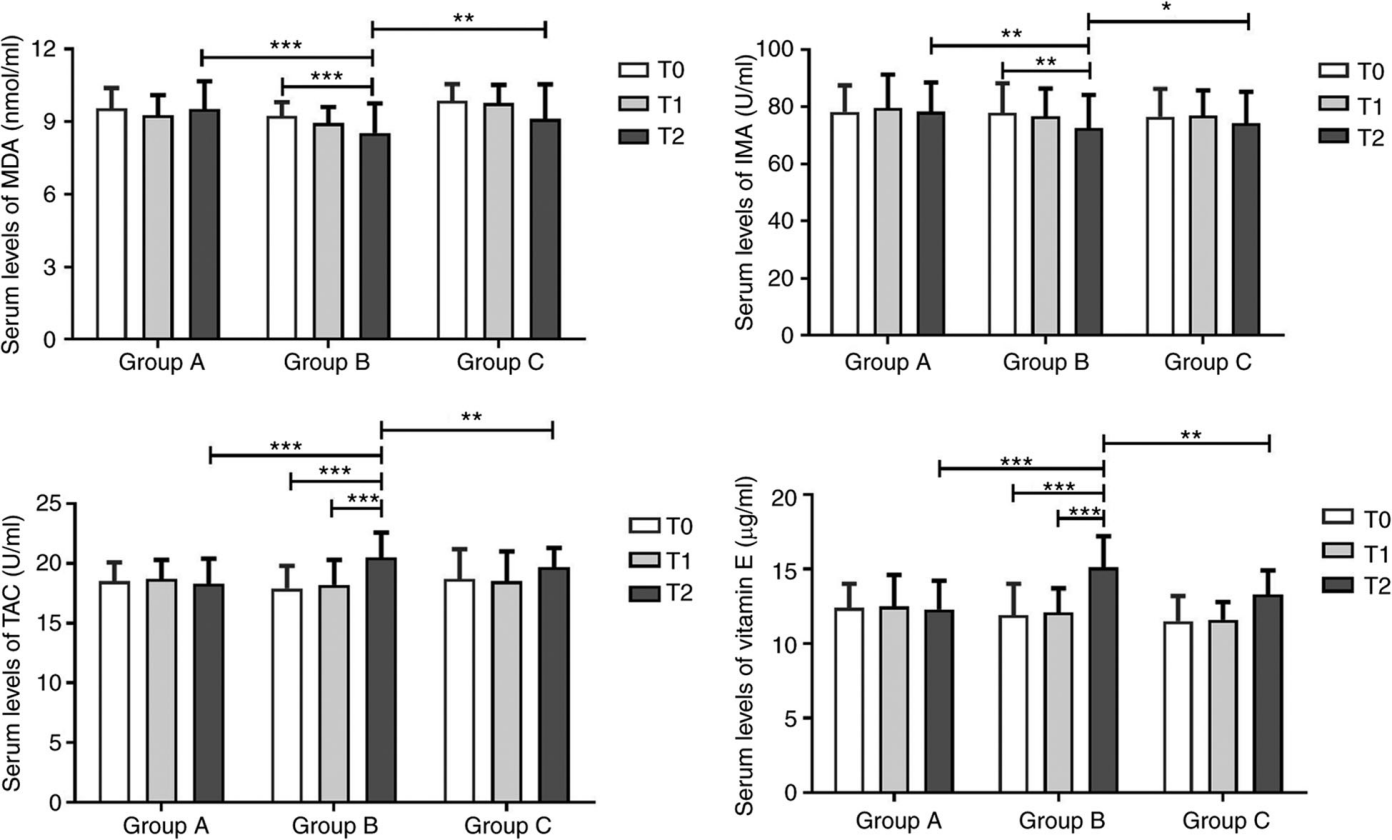
Serum oxidative stress of PCOS women in ovulation induction with or without vitamin E. *P < 0.05

The levels of four oxidative stress markers did not differ significantly among the three groups on T0 and T1 points (*P* > 0.05). Intergroup analysis demonstrated that serum concentrations of MDA (8.54 ± 1.22 nmol/L vs. 9.53 ± 1.13 nmol/L and 9.12 ± 1.42 nmol/L, both *P* < 0.05) and IMA (70.56 ± 8.21 U/ml vs. 78.25 ± 9.62 U/ml and 76.25 ± 8.02 U/ml, both *P* < 0.05) were significantly lower in the Group B compared with the Group A and C on T2. The serum levels of TAC (21.53 ± 2.13 U/ml vs. 18.32 ± 2.14 U/ml and 19.74 ± 1.62 U/ml, both *P* < 0.05) and vitamin E (15.12 ± 2.12 μg/ml vs. 12.32 ± 1.93μg/ml and 13.31 ± 1.64 μg/ml, *P* < 0.05) were significantly higher in Group B in comparison of Group A and C. In Group B, the serum concentrations of MDA (8.24 ± 0.84 nmol/L vs. 9.14 ± 0.58 nmol/L, *P* < 0.05) and IMA (70.56 ± 9.62 U/ml vs. 77.87 ± 8.54 U/ml, P < 0.05) on T2 point were significantly reduced compared with T0 point. The serum levels of TAC (21.53 ± 2.13 U/ml vs. 17.9 ± 1.9 U/ml and 18.2 ± 2.1 U/ml, *P* < 0.05) and vitamin E (15.1 ± 2.1 μg/ml vs. 11.9 ± 2.1 μg/ml and 12.1 ± 1.6 μg/ml, *P* < 0.05) on T2 point were significantly increased compared with T0 and T1 points (Fig. 2).

## Discussion

This study was conducted to assess the therapeutic effect of short-term vitamin E supplementation in the ovulation induction in PCOS women. In our study, we presented evidence that a short-term supplementation of vitamin E, an antioxidant drug, improved oxidative stress, decreased HMG dosage and increased endometrium thickness and E2 level in women with infertility and PCOS. However, reduced requirement of HMG did not reduce the number of dominant follicles compared with those without vitamin E. PCOS is one of the well-established risk factors that can increase the risk of ovarian hyperstimulation syndrome (OHSS) in controlled ovarian stimulation [22], which in some cases can be life-threatening. The possible prevention strategy includes reduction of exogenous gonadotrophin dosage and avoidance of an exaggerated response in ovarian stimulation [23], especially in controlled ovarian hyperstimulation of in vitro fertilization embryo transfer. The principles of PCOS therapies require the induction of regular uni-follicular ovulation, whilst trying to minimize the risks of OHSS and multiple pregnancy. The current study suggests that supplementation of vitamin E in PCOS resulted in a similar ongoing pregnancy rate, lower gonadotrophin requirement and possibly reduced OHSS risk. Vitamin E might be a choice for PCOS women undergoing ovarian induction.

However, we found no differences in the pregnancy rates among the three groups in our study which contrasted to a previous report [24]. In Cicek et al. [24] study, they found vitamin E administration improved the endometrial response in unexplained infertile women via the likely antioxidant and the anticoagulant effects. They enrolled unexplained infertility women without PCOS, which might contribute to the discrepancy with our study. A meta-analysis that analysed a total of 28 randomized controlled trials involving 3548 women found that antioxidants are not associated with an increased live birth rate or clinical pregnancy rate [25]. In our study, we choose a short-term of vitamin E administration at a dose of 100 mg/day based on our previous clinical medication experience. To our knowledge, there is little research on vitamin E supplement alone on PCOS patients. One double blinded randomized placebo-controlled trial investigated a combination of vitamin E (400 mg/day) and D(350,000 IU/one in 2 weeks) supplementation in the intracytoplasmic sperm injection outcomes of PCOS subjects, however, it did not add clinical support to the evidence that vitamins E and D3 might play a role in the success rate of in-vitro fertilization via an antioxidant mechanism [26]. Another study suggested that omega-3 fatty acids(1000 mg/day) and vitamin E(400 IU/day) co-supplementation for 12 weeks in PCOS women significantly improved gene expression of Lp(a) and Ox-LDL, lipid profiles and biomarkers of oxidative stress [27]. In this study, it is interesting to find that supplementation of vitamin E in PCOS may reduce serum levels of MDA and IMA, increase serum contents of TAC, and increase endometrium thickness and E2 level on day of HCG administration. This is similar to Hashemi’s study, which reported that vitamin E supplementation for 12 weeks among women with implantation failure had beneficial effects on endometrial thickness, MDA values, and gene expression of LDLR, IL-1, and TNF-α [28].

To determine whether there is a superior treatment regimen for infertile patients, several studies have been conducted to compare the efficacy of different kinds of antioxidants in the past few years [29], but little attention has been given to female infertility [24] and none have addressed PCOS. One prospective randomized trial investigated whether a subordinate multiple micronutrient (MMN) supplementation providing folic acid, vitamin B, vitamin E, vitamin C, zinc, selenium, vitamin A and other multivitamins/minerals could benefit women with anovulatory infertility or unexplained infertility who were undergoing ovulation induction by the standard treatment regimen with CC and HMG [30]. It was observed that women on MMN supplementation had a significantly higher cumulative clinical pregnancy rate and ongoing pregnancy rate compared with those who were on folic acid alone. Variation in the types of antioxidants given indicates that we need more clinical trials to assess whether one antioxidant is better than another or whether a combinatorial regimen is superior.

Some studies suggest that PCOS may cause oxidative stress [5, 6], but the impact of oxidative stress on oocytes and the reproductive function remains unclear. Increased levels of reactive oxygen species (ROS), as a result of oxidative stress, have been believed to play a key role in the pathogenesis of PCOS. ROS may negatively affect pregnancy rates, as the ROS stimulates oocyte maturation, progesterone production and luteolysis [31]. Several key limitations of the fertilization potential of oocytes have become apparent and one of them is the exponential increase of ROS in the follicular fluid resulting in the disruption of meiotic spindle formation [32]. In this study, we found that serum levels of IMA, a novel oxidative stress marker, deceased after vitamin E supplement. IMA was generated from N-terminal structural changes in albumin triggered by ROS. It has been reported that elevations in the serum IMA levels in infertile PCOS patients may suggest a possible additional role of oxidative stress mechanisms in disease pathophysiology. IMA may influence the quality of oocytes via alterations in the balance of critical follicular fluid factors in the follicular microenvironment [33].

Some investigations have shown that afamin, characterized as a novel and stable binding protein for the antioxidant vitamin E during the menstrual cycle, exists in both plasma and follicular fluid [34, 35]. Studies utilizing afamin have implicated the role of oxidative stress in patients with PCOS. A research that compared serum afamin levels in PCOS patients with healthy controls demonstrates that afamin concentrations are significantly higher in patients with PCOS and correlate significantly with homeostatic model assessment-insulin resistance (HOMA-IR) [6]. Taken together, these studies support that an antioxidant deficiency status exits in PCOS. Therefore, the ability of antioxidant defense to ROS is important for protecting tissues from oxidative damage. Vitamin E, referred to as the plant-derived and lipid-soluble antioxidants, plays an important role in reproduction through reducing oocyte apoptosis [36], and promoting oocyte maturation, endometrium proliferation and luteal function [37].

On the other hand, vitamin E deficiency is due to impaired lipoprotein synthesis or fat malabsorption syndromes. Accordingly, antioxidants, especially vitamin E, are the most effective chain-breaking lipophilic antioxidant within biological membranes. It can prevent biological damage and possess the antioxidant property of unsaturated fatty acids which can stabilize cell membranes [38]. In one study, it was found that supplementation with vitamin E reduces the oocyte apoptosis in mice treated orally with 60 mg/kg/day nicotine [36]. The effects of antioxidants on apoptosis cell specific. Vitamin E induces significant morphological changes that are consistent with apoptosis, including chromatin condensation, nuclear shrinkage, pyknosis and increased activity of caspases 3/7 [39]. It is also found that vitamin E typically protects endothelial cells from apoptosis that is induced by significant oxidative stress at the tissue level [40].

One may speculate that receiving vitamin E for a too short term may disguise its beneficial effects to ovaries and long-term supplementation may be required for a significant cumulative effect. The present observation indicates that receiving vitamin E from follicular phase reduces more serum pro-oxidative stress factors(MDA and IMA), increases more serum anti-oxidative stress markers(TAC and vitamin E), and needs less gonadotrophin dosage compared with those without vitamin E. These data may confirm the hypothesis that vitamin E can act rapidly on early follicular development in PCOS through adjusting (sensitizing or desensitizing) the sensitivity of follicles to exogenous gonadotropin without leading to excessive follicle recruitment resulting in ovarian hyperstimulation. Vitamin E also have some direct effects on follicle development by improving in vitro maturation rates and blastocyst rates of oocytes obtained after vitrification of mouse ovarian tissue [41]. Future trials may be needed to reveal the detailed mechanisms.

## Conclusions

In conclusion, a short-term supplementation of vitamin E can improve oxidative stress, reduce exogenous HMG dosage to lower the economic cost through dampening oxidative stress. However, the supplementation does not alter the pregnancy rate in the ovulation induction cycle. Moreover, given the large variety of antioxidants that have been tried, it is difficult to assess which antioxidant is superior to another and to optimize the dosage and duration.

## Data Availability

The datasets used and/or analyzed during the current study available from the corresponding author on reasonable request.

## Abbreviations

PCOS: Polycystic ovary syndrome
CC: Clomiphene citrate
HMG: Human menopausal gonadotropin
BMI: Body mass index
WHR: Waist to hip ratio
E_2_: Estradiol
T: Androstenedione
LH: Luteinizing hormone
PRL: Prolactine
FSH: Follicle-stimulating hormone
